# Supplementary material for: Splice-Junction-Based Mapping of Alternative Isoforms in the Human Proteome
Source: Cell Rep. Author manuscript; Available in PMC 2020 Jan 15. (PMC6961840; doi:10.1016/j.celrep.2019.11.026)
Supplement: 3 [file NIHMS1546469-supplement-3.zip › DF2/PXD0010154/Lung-207-Q96K76-EIENAAEEPR.pdf]

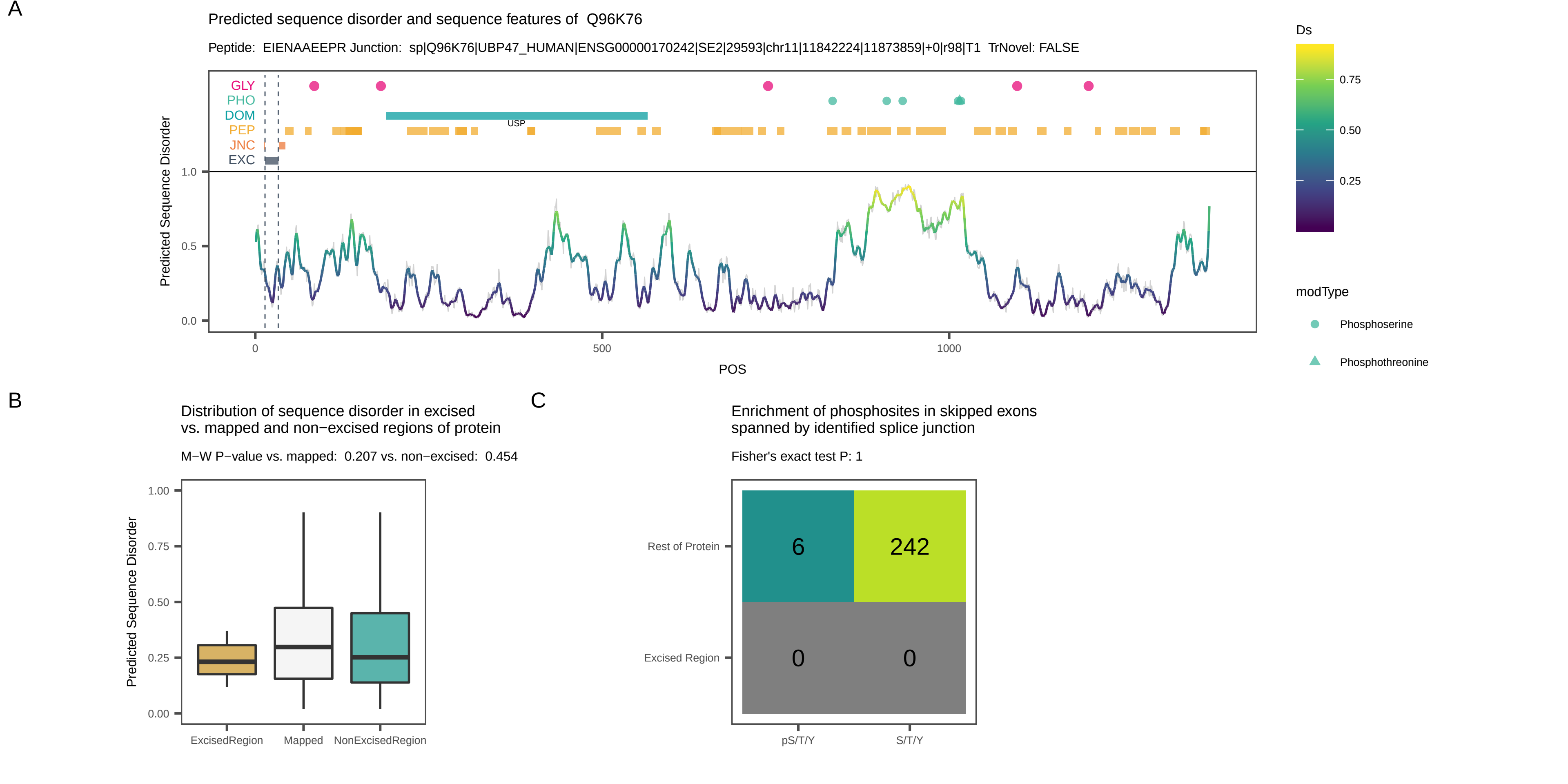
A
Predicted sequence disorder and sequence features of Q96K76
Peptide: EIENAAEEPR Junction: sp|Q96K76|UBP47_HUMAN|ENSG00000170242|SE2|29593|chr11|11842224|11873859|+0|r98|T1 TrNovel: FALSE
GLY
PHO
DOM
PEP
JNC
EXC
USP
Predicted Sequence Disorder
1.0
0.5
0.0
0
500
1000
POS
Ds
0.75
0.50
0.25
modType
Phosphoserine
Phosphothreonine
B
Distribution of sequence disorder in excised vs. mapped and non−excised regions of protein
M−W P−value vs. mapped: 0.207 vs. non−excised: 0.454
Predicted Sequence Disorder
1.00
0.75
0.50
0.25
0.00
ExcisedRegion
Mapped
NonExcisedRegion
C
Enrichment of phosphosites in skipped exons spanned by identified splice junction
Fisher's exact test P: 1
Rest of Protein
Excised Region
6
242
0
0
pS/T/Y
S/T/Y
